# Temporal trend of COVID-19 incidence and mortality rates and their relationship with socioeconomic indicators in the state of Piauí, Brazil: an ecological study, 2020-2021

**DOI:** 10.1590/S2237-96222022000200022

**Published:** 2022-09-16

**Authors:** Vanessa Rodrigues da Silva, Edildete Sene Pacheco, Osmar de Oliveira Cardoso, Luisa Helena de Oliveira Lima, Malvina Thaís Pacheco Rodrigues, Márcio Dênis Medeiros Mascarenhas

**Affiliations:** 1 Universidade Federal do Piauí, Programa de Pós-Graduação em Saúde e Comunidade, Teresina, PI, Brazil

**Keywords:** Vulnerability Analysis, COVID-19, Time Series Studies, Health Inequality Indicators, Epidemiological Monitoring

## Abstract

**Objective:**

To analyze the temporal trend of COVID-19 incidence and mortality rates and their relationship with socioeconomic indicators.

**Methods:**

This was an ecological time series study of COVID-19 cases/deaths in municipalities in Piauí, Brazil, between March, 2020 and May, 2021. Prais-Winsten linear regression model and Spearman’s correlation test were used.

**Results:**

There were 271,228 cases and 5,888 deaths in the period. There was a rising trend in COVID-19 incidence rate, while the mortality trend was stable. The spatio-temporal analyses showed higher incidence/mortality in the second and fifth quarters of the period. There was no statistically significant correlation between COVID-19 and the Social Vulnerability Index (IVS). Significant correlations between the Municipal Human Development Index (IDHM) and COVID-19 incidence (p-value < 0.001) and mortality rates (p-value < 0.001) were found.

**Conclusion:**

There was a rising trend in COVID-19 incidence and stability in COVID-19 mortality. Correlation between the MHDI and these two indicators was moderate and weak, respectively, demanding public service management decisions aimed at improving the population’s quality of life.

**Study contributions**

**Main results**

There was a rising trend in COVID-19 incidence and stability in COVID-19 mortality. There were significant correlations between the Municipal Human Development Index (IDHM) and incidence and mortality rates, but not between them and the Social Vulnerability Index (IVS).

**Implications for services**

The results can strengthen and assist the health system in formulating measures to reduce COVID-19 morbidity and mortality, as well as strategies to support the economy, aimed at the social protection of more vulnerable populations.

**Perspectives**

Articulation of actions between the various political areas and government managers, to prevent the spread of COVID-19 in successive waves and to reduce the socioeconomic consequences of the pandemic.

## Introduction

The COVID-19 pandemic, a multisystemic communicable disease caused by a highly transmissible virus (SARS-CoV-2),^1^ has become recognized as being responsible for the largest and most recent global public health crisis.^2^

Brazil had alarming COVID-19 indicators as at February 2022, when approximately 25 million cases and more than 600,000 deaths from the disease had been recorded since the pandemic began in the country.^3^ During the same period, the state of Piauí had 345,807 confirmed COVID-19 cases and 7,398 COVID-19 deaths.^3^

The marked social, economic and health service access inequalities seen between the country’s five geographic regions became more evident in the context of the pandemic in Brazil.^4^ ^,^ ^5^ These socioeconomic and health disparities, reflected in low Social Vulnerability Indices (*Índice de Vulnerabilidade Social* - IVS) and Municipal Human Development Indices (*Índice de Desenvolvimento Humano Municipal* - IDHM), have implied greater risks for the poorest populations, who have been strongly affected by the rapid and high transmission of the virus and notification of COVID-19 cases.^2^

In 2021, Tiwari et al.^2^ demonstrated that issues such as socioeconomic vulnerability, poor housing and transportation, family composition (characterized by large numbers of dependents), belonging to racial minorities, speaking English ‘less than well’, health system capacity, resources and preparedness, and epidemiological data related to cardiovascular and respiratory conditions are associated with the spread and severity of the disease.^2^ Moreover, the syndemic character of the COVID-19 pandemic has been revealed, that is, its synergistic interaction with chronic non-communicable diseases and communicable diseases, made worse by the socioeconomic context, accentuating the impacts of this global crisis.^6^

Review studies have shown that people with multiple comorbidities are the most susceptible to COVID-19 infection and have the worst prognosis.^7^ ^,^ ^8^ However, the importance of living conditions and their role in the development of the pandemic, demonstrated in social and economic indicators, have been little investigated. In addition to focusing on the problem from the commonly addressed biological perspective, the present study proposed to investigate the relationship of social aspects with COVID-19 morbidity and mortality during the pandemic, and to nurture the debate on the construction of prevention strategies, health promotion and disease control within the socioeconomic context of the population.

As such, the objective of this study was to analyze the temporal trend of COVID-19 incidence and mortality rates, and their relationship with socioeconomic indicators, in the state of Piauí, Brazil.

## Methods

This was an ecological time-series study that evaluated the COVID-19 incidence and mortality rates in the state of Piauí, from March 2020 to May 2021. The COVID-19 incidence and mortality rates were correlated to the values of two socioeconomic indicators: IVS and IDHM. 

Piauí is in Brazil’s Northeast region and, according to intercensal estimates made by the Brazilian Institute of Geography and Statistics (IBGE) for 2020, the state had a population of 3,281,480 inhabitants, distributed over 224 municipalities.^9^ In 2010, Piauí’s Human Development Index (IDH) was 0.646 and its IVS was 0.403.^10^ ^,^ ^11^

The data on COVID-19 cases and deaths were obtained from the Coronavirus Panel, a public portal updated daily by the Ministry of Health with data provided by the State Health Departments (available at https://covid.saude.gov.br/).^3^ The population information was obtained from IBGE, based on the 2010 demographic census and the estimated population projection for 2020. The IVS and IDHM scores were obtained from the website of the Institute for Applied Economic Research (*Instituto de Pesquisa Econômica Aplicada* - IPEA)^10^ ^,^ ^11^ accessed on June 1^st^, 2021.

The dependent variables were the incidence and mortality rates. The incidence rate was calculated by dividing the monthly number of confirmed COVID-19 cases in the population residing in the municipality (numerator) by the total number of the municipality’s inhabitants (denominator), multiplied by 100,000 inhabitants. The mortality rate was calculated by dividing the monthly number of confirmed COVID-19 deaths in the resident population (numerator) by the total number of the municipality’s inhabitants (denominator), multiplied by 100,000 inhabitants.

The independent variables were the IVS and the IDHM. The IVS consists of the average of the values of indicators related to three dimensions: urban infrastructure; human capital; and income and labor. The "urban infrastructure" dimension assesses access to basic sanitation and urban mobility services, while the "human capital" dimension measures individuals’ health conditions and access to education, and the "income and labor" dimension measures the financial insecurity status of those individuals.^10^ The 16 indicators that make up the IVS calculation are expressed by scores ranging from 0 to 1, where 0 corresponds to the ideal or desirable situation, and 1 corresponds to the worst situation, according to the following classification: 0-0.200 - very low IVS; 0.201-0.300 - low IVS; 0.301-0.400 - medium IVS; 0.401-0.500 - high IVS; 0.501-1.000 - very high IVS.^10^

The IDHM comprises three dimensions of human development: longevity; education and income. Such measures life expectancy at birth, access to education (average and expected years of study) and income (gross domestic product per capita); its score also varies from 0 to 1, and the closer to 1, the higher the municipality’s human development, as per the following classification: 0-0.499 - very low IDHM; 0.500-0.599 - low IDHM; 0.600-0.699 - medium IDHM; 0.700-0.799 - high IDHM; 0.800-1.000 - very high IDHM.^11^

The temporal trend analysis of the monthly COVID-19 incidence and mortality rates was performed using the linear regression method proposed by Prais-Winsten,^12^ which takes into account serial autocorrelation, i.e. the relationship between a series of values of a measurement in previous periods. We calculated monthly percent change (MPC) and its respective 95% confidence intervals (95%CI), for all the state’s municipalities. The incidence and mortality rates were categorized as follows: rising (p-value < 0.05 and positive beta); falling (p-value < 0.05 and negative beta); stable (p-value ≥ 0.05).^12^

The COVID-19 incidence and mortality rates were calculated for all 224 Piauí municipalities. However, given the high number of municipalities, only those with incidence (n = 23 municipalities) and mortality rates (n = 24 municipalities) in the highest decile of the distribution of the values are presented in Tables 1 and 2. These tables also include the averages of the incidence and mortality rates in the 1^st^ quarter (March to May 2020) and the 5^th^ quarter (March to May 2021), and the standard deviation (SD) of all those averages, corresponding to the period analyzed.

The correlations between the COVID-19 incidence and mortality rates, and the IVS and the IDHM - considering the three dimensions of each index - were analyzed using Spearman’s correlation test.^13^ Kolmogorov-Smirnov and Shapiro-Wilk tests of normality were performed, to check for non-normal distribution of incidence and mortality rates taking a p-value < 0.05 in both tests. Statistically significant (p-value < 0.05) Spearman correlation results were classified into five categories: very weak (0.00-0.19); weak (0.20-0.39); moderate (0.40-0.59); strong (0.60-0.79); very strong (0.80-1.00).^14^

We prepared maps of the distribution of the quarterly averages of the COVID-19 incidence and mortality rates in the municipalities of Piauí. The statistical analyses were performed using Stata 14 (StataCorp LP, College Station, USA, serial No. 401406370959); and the maps were produced using QGIS 3.16.

The study was conducted using anonymous secondary data obtained from public access platforms. As such the study project did not need to be submitted to a Research Ethics Committee.

## Results

In the period from March 2020 to May 2021, 271,228 COVID-19 cases were confirmed, resulting in an incidence rate of 8,265.4/100,000 inhabitants. In the same period there were 5,888 COVID-19 deaths, corresponding to a mortality rate of 179.4/100,000 inhabitants 34.8% of cases and 37.0% of deaths were registered for residents of the state capital, Teresina. By May 2021, all the municipalities in Piauí had notified cases of COVID-19 and only two had no records of COVID-19 deaths.

Table 1 shows total and quarterly incidence rates, and the temporal trend of this indicator in the 23 municipalities in the decile with the highest incidence rates; of these 23 municipalities, 20 had a rising trend. The highest incidence rates were found in the municipalities of Demerval Lobão (21,445.1/100,000 inhabitants), Lagoa do Piauí (21,055.2/100,000 inhabitants), and Lagoa do Barro do Piauí (19,222.5/100,000 inhabitants). Lagoa do Piauí had the highest average incidence rate in the 5^th^ quarter (3,846.8/100,000 inhabitants). The largest monthly percent changes were found in the municipalities of Caridade do Piauí (74.5%; 95%CI 44.0;111.6), Miguel Leão (74.3%; 95%CI 22.4;148.3) and Francisco Macedo (69.6%; 95%CI 9.0;163.9).

**Table 1:** COVID-19 incidence rate (per 100,000 inhabitants), total and quarterly (1^st^ and 5^th^ quarters), and temporal trend of the monthly incidence rate of the municipalities in the first decile of incidence, Piauí, Brazil, March/2020-May/2021

Municipalities	Incidence rate			SD^b^	MPC^c^	95%CI^d^	p-value^e^	Trend
	Total	1^st^ Q^a^	5^th^ Q^a^					
Demerval Lobão	21,445.1	106.1	3,129.0	1,212.7	57.1	5.4;134.1	0.030	Rising
Lagoa do Piauí	21,055.2	139.4	3,846.8	1,506.1	64.8	6.7;154.5	0.028	Rising
Lagoa do Barro do Piauí	19,222.5	57.3	2,305.8	904.8	52.8	4.3;123.8	0.032	Rising
Baixa Grande do Ribeiro	18,327.5	23.0	1,214.1	928.3	64.8	0.9;169.0	0.046	Rising
Uruçuí	16,107.1	94.3	1,482.8	733.1	54.3	0.8;136.3	0.046	Rising
Miguel Leão	15,378.4	0.0	3,584.8	1,460.7	74.3	22.4;148.3	0.005	Rising
Bertolínia	15,089.9	24.2	1,502.8	883.6	59.6	1.5;150.8	0.044	Rising
Piripiri	14,811.8	42.4	2,614.2	965.1	63.2	11.4;139.2	0.016	Rising
Landri Sales	13,836.8	25.2	3,575.7	1,499.5	68.6	44.6;96.6	< 0.001	Rising
Francisco Macedo	13,593.8	0.0	1,151.6	511.0	69.6	9.0;163.9	0.023	Rising
Monsenhor Gil	13,290.4	60.0	1,905.8	754.9	57.9	4.7;138.1	0.032	Rising
Antônio Almeida	12,712.9	0.0	590.0	840.9	42.3	-13.6;134.5	0.151	Stationary
Hugo Napoleão	12,632.1	25.8	851.2	873.0	54.1	-2.9;114.4	0.064	Rising
Santa Cruz do Piauí	12,454.0	5.3	187.0	991.7	39.5	-20.7;145.5	0.225	Stationary
Floriano	12,204.9	42.8	1,048.2	448.6	57.8	4.1;139.2	0.034	Rising
Belém do Piauí	12,182.9	56.1	645.0	797.6	50.5	9.0;107.6	0.017	Rising
Bom Jesus	12,155.8	78.6	1,081.6	491.8	60.5	2.9;150.2	0.039	Rising
José de Freitas	11,986.5	23.0	2,335.4	900.3	65.7	15.3;138.0	0.010	Rising
Morro do Chapéu do Piauí	11,466.7	117.7	1,324.3	578.1	48.1	-1.6;123.0	0.059	Stationary
Caridade do Piauí	10,953.8	0.0	1,508.6	842.4	74.5	44.0;111.6	< 0.001	Rising
Oeiras	10,931.6	54.0	1,356.6	491.0	59.1	7.5;135.4	0.020	Rising
Teresina	10,872.9	86.6	1,246.7	420.1	51.6	5.4;118.0	0.030	Rising
Guadalupe	10,793.6	3.2	1,362.0	725.9	57.8	15.6;115.3	0.010	Rising

a) Q: Quarter; b) SD: Standard deviation; c) MPC: Monthly percent change; d) 95%CI: 95% Confidence interval; e) Prais-Winsten linear regression.

Table 2 shows the total and quarterly mortality rates, and the temporal trend of this indicator in the 24 municipalities in the decile with the highest COVID-19 mortality rates; of these 24 municipalities, ten had a rising trend. The highest mortality rates were found in the municipalities of Água Branca (354.9/100,000 inhabitants), Beneditinos (324.6/100,000 inhabitants) and Antônio Almeida (315.5/100,000 inhabitants). The highest monthly percent changes were found in the municipalities of Simplício Mendes (40.7%; 95%CI 26.3;56.7) and Eliseu Martins (36.6%; 95%CI 19.1;56.6); Simplício Mendes was the municipality with the highest average COVID-19 mortality rate in the 5^th^ quarter (83.9/100,000 inhabitants).

**Table 2:** COVID-19 mortality rate (per 100,000 inhabitants), total and quarterly (1^st^ and 5^th^ quarters), and temporal trend of the monthly mortality rate of the municipalities in the first decile of mortality, Piauí, Brazil, March/2020-May/2021

Municipalities	Mortality rate			SD^b^	MPC^c^	95%CI^d^	p-value^e^	Trend
	Total	1^st^ Q^a^	5^th^ Q^a^					
Água Branca	354.9	11.5	24.9	17.0	9.2	-17.0;43.6	0.502	Stationary
Beneditinos	324.6	0.0	54.1	21.1	30.2	7.2;58.1	0.012	Rising
Antônio Almeida	315.5	0.0	21.1	24.7	17.8	-9.8;53.7	0.208	Stationary
Simplício Mendes	306.0	0.0	83.9	35.7	40.7	26.3;56.7	< 0.001	Rising
Valença do Piauí	296.2	1.6	54.2	20.5	29.0	17.7;41.4	< 0.001	Rising
Monsenhor Gil	293.5	0.0	44.2	16.8	27.0	5.1;53.5	0.017	Rising
Eliseu Martins	284.0	0.0	67.8	28.1	36.6	19.1;56.6	< 0.001	Rising
Piripiri	282.2	1.0	57.5	22.2	27.7	10.8;47.1	0.003	Rising
Campo Maior	270.8	2.1	45.6	18.1	19.9	-2.8;48.0	0.085	Stationary
Parnaíba	260.6	2.0	48.1	19.8	30.1	-5.3;78.7	0.097	Stationary
Piracuruca	259.8	2.3	60.2	24.2	23.8	9.1;40.5	0.003	Rising
Teresina	251.0	3.3	32.0	13.9	32.9	-0.5;77.4	0.053	Stationary
Marcolândia	245.9	0.0	35.3	14.5	15.4	-6.6;42.7	0.167	Stationary
Nossa Senhora de Nazaré	245.4	0.0	34.2	12.4	24.8	-0.6;54.9	0.056	Stationary
Pavussu	245.3	9.1	54.4	22.6	22.4	-3.9;55.9	0.095	Stationary
Cocal de Telha	245.0	0.0	40.9	15.7	25.5	0.5;56.8	0.046	Rising
Lagoinha do Piauí	245.0	0.0	35.1	13.4	15.4	-5.1;40.3	0.137	Stationary
Manoel Emídio	243.0	6.2	37.4	14.3	7.6	-15.5;37.1	0.524	Stationary
Uruçuí	235.5	3.1	23.2	11.1	15.3	-5.5;40.6	0.147	Stationary
Cajueiro da Praia	234.6	0.0	21.8	11.4	13.2	-14.7;50.2	0.360	Stationary
Passagem Franca do Piauí	231.3	0.0	30.9	14.5	12.3	-11.1;42.0	0.300	Stationary
Santa Cruz do Piauí	224.1	0.0	5.3	12.8	6.3	-19.7;40.7	0.650	Stationary
Várzea Branca	222.8	0.0	40.4	16.1	20.9	0.6;45.4	0.040	Rising
Floriano	221.6	1.1	33.4	12.9	22.4	2.6;45.9	0.030	Rising

a) Q: Quarter; b) SD: Standard deviation; c) MPC: Monthly percent change; d) 95%CI: 95% Confidence interval; e) Prais-Winsten linear regression.

In the period studied, the incidence rate temporal trend was rising in 146 municipalities, while mortality rates were stable in 150 municipalities in the state of Piauí (Tables 1 and 2).

**Figure 1:**
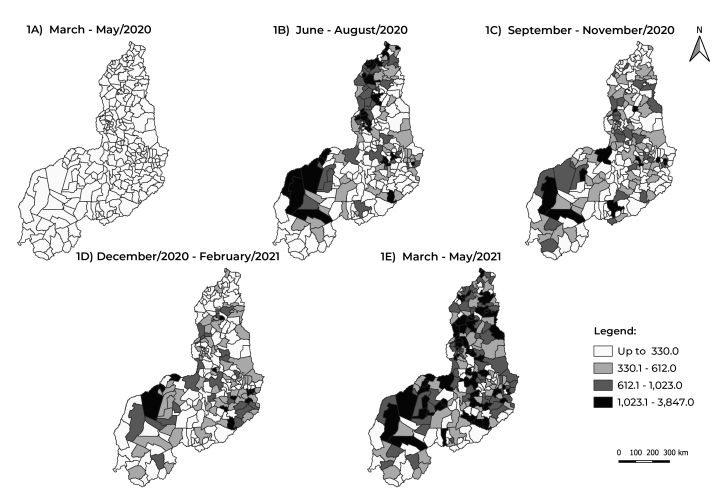
Evolution of the quarterly average COVID-19 incidence rate (per 100,000 inhabitants), by municipality of residence, Piauí, Brazil, March/2020-May/2021

Figure 1 shows the geographical distribution and the evolution of the quarterly averages of the COVID-19 incidence rates in the state’s 224 municipalities. In the 1^st^ quarter, all the municipalities reported incidence rates lower than 330.2/100,000 inhabitants (Figure 1A), followed by a sharp increase in the 2^nd^ and 5^th^ quarters, with incidence rates higher than 1,023.1/100,000 inhabitants, which represents increases in COVID-19 incidence in the order of 13.8% and 25.0%, respectively (Figures 1B and 1E).

**Figure 2:**
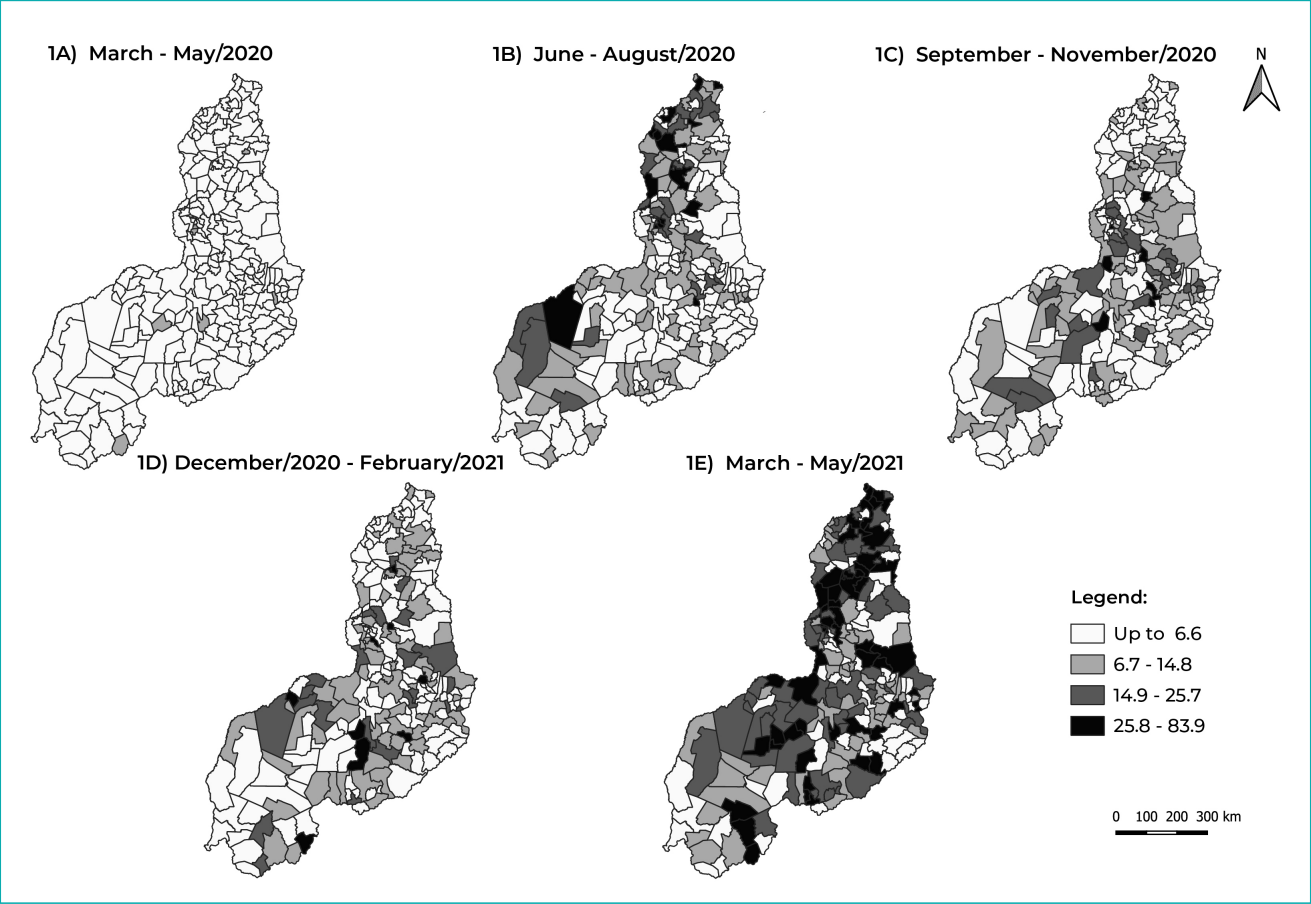
Evolution of the quarterly average COVID-19 mortality rate (per 100,000 inhabitants), by municipality of residence, Piauí, Brazil, March/2020-May/2021

Figure 2 shows the evolution of the quarterly averages of the COVID-19 mortality rate. There was an increase in the 2^nd^ and 5^th^ quarters, when 6.3% and 25.0% of the municipalities, respectively, had rates higher than 25.7/100,000 inhabitants (Figure 2B and 2E). In the 1^st^ quarter, most municipalities had mortality rates of up to 6.6/100,000 inhabitants (Figure 2A).

There were no statistically significant correlations between COVID-19 incidence and the IVS (r = -0.049; p-value = 0.465), or between COVID-19 mortality and the IVS (r = -0.110; p-value = 0.099). In relation to the IDHM, a moderate and weak correlation was observed, respectively, with the incidence rate (r = 0.419; p-value < 0.001) and the mortality rate (r = 0.358; p-value < 0.001) (Table 3).

**Table 3:** Correlation between COVID-19 incidence and mortality rate indicators (per 100,000 inhabitants) and socioeconomic indicators, Piauí, Brazil, March/2020-May/2021

Socioeconomic indicators	Incidence rate		Mortality rate	
	r^a^	p-value^b^	r^a^	p-value^b^
**IVS^c^**	-0.049	0.465	-0.110	0.099
Urban infrastructure	0.076	0.258	0.067	0.315
Human capital	-0.132	0.048	-0.167	0.012
Income and labor	-0.196	0.003	-0.288	< 0.001
**IDHM^d^**	0.419	< 0.001	0.358	< 0.001
Longevity	0.151	0.024	0.270	< 0.001
Education	0.373	< 0.001	0.274	< 0.001
Income	0.449	< 0.001	0.385	< 0.001

a) R: Correlation coefficient; b) Spearman’s correlation test; c) IVS: Social vulnerability index; d) IDHM: Municipal human development index. Note: "c" and "d" correspond to the total value, considering the three dimensions.

Considering the three IVS dimensions, no correlation was found between urban infrastructure and incidence rates (r = 0.076; p-value = 0.258) and mortality rates (r = 0.067; p-value = 0.315). Correlations, ranging from very weak to weak, were found between the "human capital" dimension and the incidence rate (r = -0.132; p-value = 0.048) and the mortality rate (r = -0.167; p-value = 0.012), and between the "income and labor" dimension and the incidence rate (r = -0.196; p-value = 0.003) and the mortality rate (r = -0.288; p-value < 0.001). As for the IDHM, correlation was identified between all dimensions (longevity, education and income) and the incidence/mortality rates in all the municipalities analyzed, with income and incidence rate standing out (r = 0.449; p-value < 0.001 - effect magnitude: moderate) (Table 3).

## Discussion

Most of the Piauí municipalities analyzed, i.e. those with the highest COVID-19 incidence and mortality rates, showed a rising incidence rate trend and a stationary mortality rate trend between March 2020 and May 2021. Furthermore, as shown in the maps illustrating this analysis, there was a significant increase in incidence and mortality rates in all the state’s municipalities throughout the period studied. Furthermore, the analysis of all municipalities did not reveal strong correlations of incidence and mortality rates with the IVS, although significant correlations were found between incidence and mortality rates and the IDHM.

In the light of the COVID-19 scenario in Brazil as a whole, Piauí has particularities with regard to the IVS, reflected in COVID-19 morbidity and mortality and in how the pandemic was addressed in the political, social and economic spheres. These specificities can be related to 186 municipalities (83%) being classified as having high or very high social vulnerability, according to the Fundação Centro de Pesquisas Econômicas e Sociais do Piauí (CEPRO).^15^ Moreover, only three municipalities in the state are classified as having low social vulnerability, Teresina, Picos and Floriano; none of the municipalities had a very low IVS.^15^

The state capital city, Teresina, registered the highest number of confirmed COVID-19 cases and deaths in the state. This result corroborates data from a study conducted in the first 60 days following the emergence of the disease in Piauí,^16^ when Teresina, precisely the municipality most affected in that period, accounted for 54.4% of COVID-19 cases and 47.1% of COVID-19 deaths in the state. This finding regarding Teresina can be attributed to the fact that the city is the most populated in the state and has a numerous road and airway connections with other regions of the state.^17^ Furthermore, Piauí’s high complexity health services are concentrated in Teresina, causing an imbalance in service availability as well as overloading the health system in the capital.^17^ ^,^ ^18^

Teresina, Floriano, Campo Maior, Uruçuí and Valença are among the cities with stable mortality rates. They have a more structured health care network, either with regard to primary care, responsible for detecting cases and vaccinating against COVID-19, or with regard to the hospital network present in these cities, the services of which are better able to treat severe cases of the disease.^19^

Some of the municipalities evaluated did not show statistical significance in the temporal trend analysis, for example, Morro do Chapéu do Piauí, with regard to the incidence rate evaluation, and Campo Maior, Teresina, Nossa Senhora de Nazaré, Pavussu, Lagoinha do Piauí, and Uruçuí with regard to the mortality rate evaluation. However, these municipalities showed a rising trend that, for state planning purposes, needs to be taken into consideration when formulating measures intended to ensure stability or reduction in the number of cases and deaths.

After the first cases of COVID-19 were registered in Piauí, social distancing measures, such as the closure of schools and non-essential establishments, were implemented in a decentralized and independent manner by the municipalities, which chose to employ their own intervention measures, since there was no single recommendation from the federated entity.^20^ Prevention measures were relaxed when there was a decrease in the number of cases and deaths, especially in late 2020 and early 2021, these being periods within the 3^rd^ and 4^th^ quarters evaluated. This was followed by an increase in COVID-19 morbidity and mortality in the state, with the occurrence of “waves” or “peaks” of the pandemic.^21^

As also happened nationwide,^22^ two “waves” of the disease occurred during the period analyzed in this study: the first, from June to August 2020; and the second, from March to April 2021. The increase in morbidity and mortality noted during the second wave was greater than that seen in the first wave, both in Piauí and in the rest of the country, since the states adopted preventive measures before and during the first wave of COVID-19,^22^ whereas in the second wave, health service managers delayed the implementation of strict distancing measures, using them as a last resort in the face of imminent increased morbidity and mortality.^22^

Unlike the nationwide study developed by Martins-Filho et al.,^14^ the results of our study showed no correlation between COVID-19 morbidity and mortality and the IVS. 

Piauí has one of Brazil’s poorest Human Development Indices, coming in fourth place only after the states of Pará, Maranhão and Alagoas.^23^ None of the Piauí municipalities has an IDHM falling in the "very high" category (above 0.800), and only Teresina and Floriano are classified as having a high IDHM (between 0.700 and 0.799).^11^ The correlations identified between the IDHM and COVID-19 morbidity and mortality, with weak or moderate magnitudes, corroborate the results of a similar analysis carried out in the state of Ceará.^24^ The authors of that study concluded that Ceará’s municipalities that have a higher IDHM also have higher SARS-CoV-2 circulation and greater COVID-19 transmissibility, since municipalities in the state’s interior regions continue to have intense social and economic relations with the largest urban centers, due to the great mobility of the population in search of services that those centers offer.^24^ Given the similar relationships existing between Piaui’s municipalities, this argument may well be valid for Piauí as well.

Certain limitations need to be taken into consideration when interpretating the data presented. These include the delay in the notification of COVID-19 deaths on the Mortality Information System (SIM) in the period in which the data was collected. As such, we chose to use the Coronavirus Panel, considered to be the official information channel on COVID-19 in Brazil and which, despite its weaknesses, is easy to handle and makes data available quickly.^25^

Furthermore, we highlight the lack of up to date socioeconomic data for analyzing their association with incidence and mortality rates, since the 2020 census was not carried out and, therefore, the associations found may not reflect current reality. The low number of rapid tests and COVID-19 case and death underreporting must also be considered, since this can lead to the indicators calculated being underestimated and makes it difficult to achieve a reliable understanding of the epidemiological analysis of COVID-19 in Piauí. These limitations are inherent to the surveillance systems used, to the Federal Administration, and to the performance of the Piauí health service management, thus limiting researchers’ power of intervention to remedy them.

The absence of strong correlations between the morbidity and mortality indicators and the IVS are cause for concern. They demonstrate the spread of COVID-19, in both the most vulnerable and also in the least vulnerable social classes. Moreover, the significant correlations between these incidence and mortality rates and the IDHM point to the need for policies that ensure the population’s development free from significant impacts, both in the current and future health crises, considering the three IDHM dimensions - longevity, education, and income.

In view of a country so diverse, of continental dimensions and acknowledged inequalities as Brazil, there is a set of strategies to be considered. The diversity of the territory requires the Federal Administration to play an active role as well as intense cooperation between state and municipal governments. Among the joint actions necessary to enhance the State’s response to the pandemic are: (i) transparent and detailed monitoring of the epidemiological situation; (ii) ensuring social distancing; (iii) articulating communication between the various groups in society; (iv) strengthening both the public health system, at its various levels, and the private health system; (v) and measures to support employment and the economy, with strategies aimed at the social protection of more vulnerable populations. In view of this, in Piauí and other states, actions must also be articulated, which requires coordination between the various areas of government policy and management, to avoid the spread of the disease in successive waves and reduce its socioeconomic consequences.^26^

This study used an innovative approach, rarely seen before in the literature, by analyzing COVID-19 incidence and mortality rates using the IVS and IDHM. By confirming the increase of confirmed COVID-19 cases in the state’s municipalities, to the extent that there is a correlation between COVID-19 and the IDHM, this study both helps and demands sensitivity from all public service administrators in the use of management tools to address the pandemic. Finally, further research and publications on the subject are recommended, as well as the adoption of measures that take into account socioeconomic aspects, in order to reduce morbidity and mortality and the impacts caused by the COVID-19 pandemic in the population. 
